# Approach to theoretical perspectives of “sexual harassment”: review and bibliometric analysis from social sciences

**DOI:** 10.3389/fpsyg.2023.1088469

**Published:** 2023-05-18

**Authors:** Cristina Cuenca-Piqueras, Juan Sebastián Fernández-Prados, María José González-Moreno

**Affiliations:** Department of Sociology, CEMyRI, University of Almería, Almeria, Spain

**Keywords:** sexual harassment, bibliometric analysis, literature review, theoretical models, gender studies

## Abstract

The main goal of this paper is to give an overlook of the current state of sexual harassment. In order to do so, we started making a synthesis of the main theoretical approaches to explain sexual harassment, trying to group the studies into different currents that have historically analyzed this type of violence, to see whether the bibliometric analysis shows a similar presence of the different approaches. To carry out the bibliographic analysis we extracted the documents from the Scopus databases (using the keyword “sexual harassment” up to the year 2021 in the field of social sciences), where after these texts were examined with the tool VOSviewer. A description was made of the evolution of the number of articles on sexual harassment and within the areas of research and, also, the coincidences of keywords and co-authorships, highlighting the nationality and the main authors in number of citations. The results show a growth in interest in researching sexual harassment, specifically after the allegations against producer Harvey Weinstein and the #metoo movement. In the keyword analysis, there is a trend towards studies focused on the work environment and with a gender perspective. Finally, in the cluster analysis of the authorship of the texts, the results suggest the different theoretical approaches most used in the analysis of sexual harassment: socio-cultural, organizational, and multi-dimensional.

## 1. Introduction: theoretical framework

The study of sexual harassment began in the US in the 1970s. This first organized resistance to this phenomenon emerged at the intersection of two forms of activism: the movements against discrimination in the workplace and the feminist opposition to violence against women. It was in these early moments when people began to question why the behaviors that made women uncomfortable in their workplaces were happening, and to try to raise awareness about them. Thus, these first approaches try to extrapolate feminist theories on rape and domestic violence to articulate explanations of sexual harassment (Baker, 2007).

In this overview, we mainly focus on the primary approaches, composed with the help of great amounts of research material, which classify the different theoretical approaches (O’Donohue et al., 1998). The most used approach by researchers is the socio-cultural theoretical model. This theory postulates that sexual harassment is a product of culturally legitimized differences in power and status between men and women (MacKinnon, 1979). This model highlights the origins of sexual harassment in patriarchal society and is perceived as a way used by men to control and dominate women, at work as well as in society. Indeed, it should be considered that most of the research on sexual harassment has been based on this model, so it can be considered the hegemonic theoretical line. One of the most frequent criticisms of this model is that it does not address the factors related to the work environment. It is considered essential to take into account these factors in order to understand why sexual harassment occurs (Welsh, 1999, p. 176).

The second model analyzes sexual harassment from the organizational point of view (Chamberlain et al., 2008; McDonald, 2012; Minnotte and Legerski, 2019). This approach considers the role of the work environment in sexual harassment. Specifically, it analyses certain organizational characteristics related to power, and how they affect the rates of this kind of violence (Fitzgerald et al., 1997; O’Donohue et al., 1998).

Finally, we present another approach in more detail. Even though there are many names for the “multidimensional model,” we use this term, following Fitzgerald and Buchanan (2008). In this approach, the authors try to close the gap that exists in ethnocentric research. They consider that there have been no studies that have examined how an individual’s sex and ethnicity might jointly affect his or her experiences of both types of harassment at work. Studies of sexual harassment have focused on women’s experiences and have led to the development of theories and measures that are largely based on White women’s experiences and that overlook or even exclude those of minority women (Buchanan and Ormerod, 2002; Shupe et al., 2002; Cortina and Wasti, 2005; Berdah and Moore, 2006). Nowadays, the “multidimensional model” focused on the importance of intersectional analysis can be considered as one of the future lines of research (Cortina and Areguin, 2021). The lack is present both in workplace sexual harassment analyzes and in educational contexts of sexual harassment (Bondestam and Maja Lundqvist, 2020).

At present, interest in the study of sexual harassment has increased considerably. One turning point could be when the descriptions of Harvey Weinstein’s victims appeared in the New York Times (Ronan, 2017), and after the creation and rise in popularity of the hashtag #MeToo. In this sense, the visibilization work done by actresses should be emphasized, using the movement, but, also through their actions in award ceremonies, with the “Time’s up” initiative, where an important number of actresses claimed equality in the workplace and the visibilization of sexual harassment, especially in the entertainment industry (Cobb and Horeck, 2018). Awareness at a global level was made evident during 2018, culminating, at the media level, with the naming of the person of the year by Time magazine to the #metoo movement, describing them as “the silence breakers.” Therefore, the social change in the perception of these behaviours or the so-called “Weinsten Effect” is clear at a global level, but it does not have the same intensity in all countries, and other environmental and political factors may also have an impact. In this sense, issues such as regulatory changes (passing of innovative laws on sexual offences), previous feminist debates on harassment, consent or sexual coercion and very shocking cases (such as gang rapes), are issues that could mean differences between some countries and others (Hörnle, 2018).

Taking these considerations into account, we agree with Lengnick-Hall opinion: “what we do not know about sexual harassment far exceeds what we do know” which considers that there are still many issues related to sexual harassment that are still not explained (Lengnick-Hall, 1995, p. 84). In short, it seems to us very relevant to continue analysing this topic and to advance in our knowledge of it. This contribution will focus on specifying the aspects that have already been analysed and the gaps in the research.

## 2. Method

### 2.1. Methodology

The goal of this research is the scientific production around “sexual harassment” in the field of social sciences. The type of analysis that will be carried out is exploratory, descriptive and quantitative, based on the techniques and tools of bibliometric analysis of the documents stored in the Scopus bibliographic database. Systematic literature reviews in research studies are very useful for researchers because they help them go deeper into the field of study, get to know the most cited trends, networks, works and authors, and to raise questions for future research (Muyor Rodríguez and Fernández-Prados, 2021). In the same way, the review has followed the main criteria of the Prisma methodology checklist: the explicit inclusion of review in the title, the design of a flow chart, etc. (Page et al., 2021).

To these advantages, we can add some risks such as “inflated” citations due to too much self-citation, distinguishing citations to criticize a certain work, defining precise semantic search fields, etc. (Holden et al., 2005). In any case, bibliometric studies use a formal and rigorous procedure that guarantees the quality of the results obtained by employing increasingly sophisticated techniques and analysis software and increasingly systematic and complete compilations of scientific publications (Ball, 2021).

### 2.2. Sample

The steps followed to delimit the scope of the study were based on the decision made in the consulted database, the search term, and the fields, as well as the time interval, in which the sample of documents to be analyzed would finally be located. First, Scopus was chosen because it is the largest database of abstracts and citations of peer-reviewed literature, containing about 25,100 indexed titles including journals, books, proceedings, etc., and about 77.8 million records and documents. Further, typically Scopus had the highest coverage of most disciplines in the social sciences while WoS had the least (Stahlschmidt and Stephen, 2020).

Secondly, the search term selected aims to collect publications that address the topic or term “sexual harassment,” that appear in the title, abstract or keyword fields, finding about 8,827 documents. In addition, the search was limited to articles published up to and including the year 2021, excluding those published in 2022 and those announced for 2023, as these years are still incomplete, leaving 8,000 documents. Finally, records limited to the thematic area of Social Sciences were selected, reducing the final sample to 3,384 documents.

**Figure 1:**
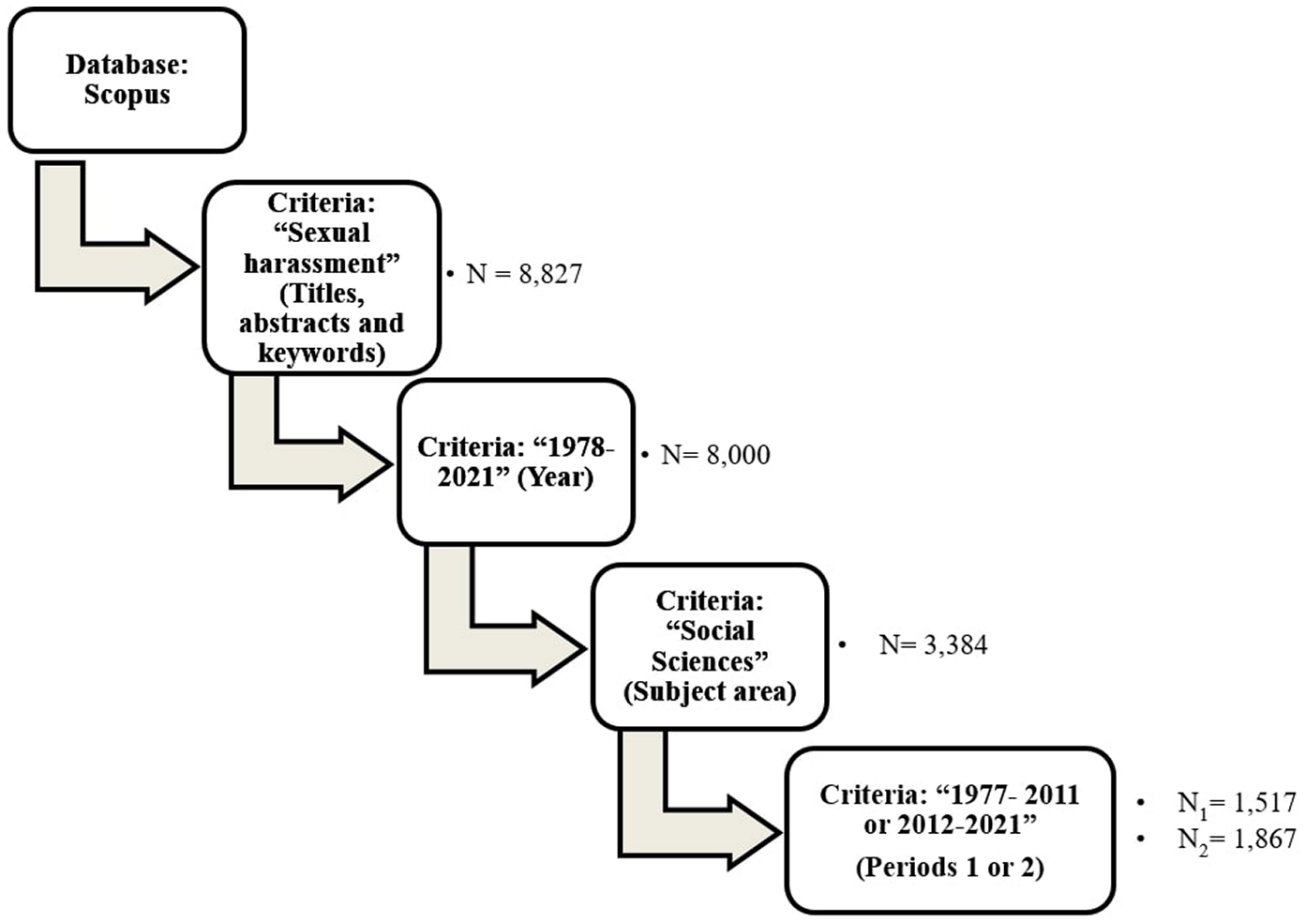
Data selection and analysis step-by-step.

In summary, these three steps have been followed as a flowchart for making decisions about the final sample: (a) TITLE-ABS-KEY (“sexual harassment”) = 8,827 document results; (b) TITLE-ABS-KEY (“sexual harassment”) AND (EXCLUDE (PUBYEAR, 2023) OR EXCLUDE (PUBYEAR, 2022)) = 8,000 document results; (c) TITLE-ABS-KEY (“sexual harassment”) AND (EXCLUDE (PUBYEAR, 2023) OR EXCLUDE (PUBYEAR, 2022)) AND (LIMIT-TO (SUBJAREA, “SOCI”)) = 3,384 document results. In addition, to detect trends in the analysis we have divided the final sample into two periods or halves from the beginning (1978) to 2011 and from 2012 to 2021 (see Figure 1).

## 3. Results

### 3.1. Descriptive

The first published article containing “sexual harassment” in its title, abstract or keywords is dated in 1978. Since then, the number of publications has continued to grow, although the last 5 years have accounted for more than one-third (38%), and the last decade for slightly more than half (55.1%). This first descriptive analysis of the evolution of publications led us to compare different bibliometric aspects between the results of the last decade and those of the previous decades.

The analysis of the areas of knowledge shows the prominent role of Psychology in the scientific production about sexual harassment with 20.5% of the documents published. It is also worth emphasizing three other areas as Arts and Humanities; Business, Management and Accounting; and Medicine, which each exceed 10 % of the documents. The evolution of the relative weight of the disciplines indicates that in the last decade Psychology has declined significantly (from 25.9% in the period 1978–2011 to 16.6% in the last 10 years) diversifying the number of areas involved in scientific publications on the subject of sexual harassment.

Most of the documents were written by authors of US nationality (54.1%), although in the last decade this nationality has not reached half of the number of publications (47.4%). This relative decrease in the number of works written by North Americans has been offset by the increase in contributions from other English-speaking countries such as the United Kingdom and Australia. However, we should highlight the exponential increase of countries such as Spain, which has gone from contributing with 0.3% of the documents before 2012 to a 3% in the last decade, India from 0.9 to 4.1% or Sweden from 0.6 to 2.2%. Once again, the diversity in the origin of the authors is another trend in the scientific production in the field of sexual harassment.

### 3.2. Keywords and co-occurrences analysis

The 3,384 documents contain 5,770 different keywords and as expected, the predominant one is “sexual harassment” which, moreover, has significantly increased its appearance in the last decade, reaching almost half (45.2%) of the cases (see Table 1). This means that sexual harassment used to appear “secondarily” in the abstracts, but in the most recent publications, it has acquired greater relevance. Other prominent keywords are “human” and “female,” which have increased somewhat in relative weight in recent times. However, other keywords have grown dramatically such as “sexual violence” (from 0.3% between 1978 and 2011 to 6.4% between 2012 and 2021) and “psychology” (from 0.7 to 6.7%).

**Table 1:** Evolution of keywords by periods.

Keyword	1978–2021	1978–2011	2012–2021
Sexual harassment	40.6%	34.9%	45.2%
Human	18.5%	17.3%	19.5%
Female	15.3%	14.4%	16.1%
Article	12.8%	15.6%	10.5%
Male	12.5%	11.7%	13.2%
Humans	12.0%	11.3%	12.6%
Adult	9.5%	8.1%	10.6%
Gender	7.4%	4.5%	9.6%
United States	5.4%	7.1%	4.0%
Questionnaire	4.3%	4.3%	4.4%
Adolescent	4.3%	3.4%	5.0%
Psychology	4.0%	0.7%	6.7%
Sexual violence	3.7%	0.3%	6.4%
Workplace	3.7%	3.0%	4.2%
Violence	3.4%	3.3%	3.5%
Major clinical study	3.4%	2.6%	4.0%

**Figure 2:**
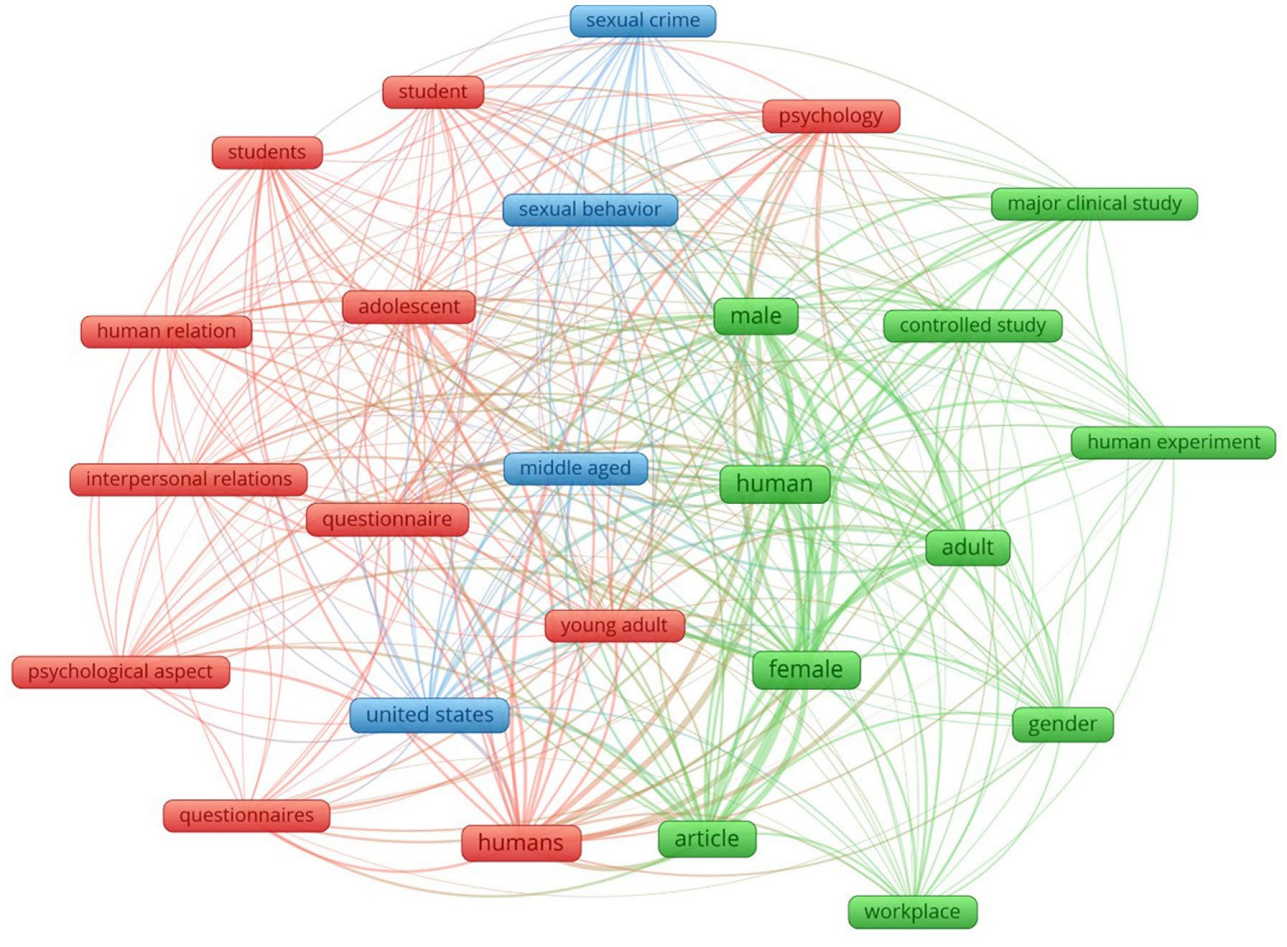
Analysis of co-occurrence among 25 keywords most frequency.

Figure 2 shows the co-occurrences of keywords in the same document using the VOSviewer software, eliminating the word “sexual harassment” to avoid distortions due to its obviousness and excessive weight that would overshadow the rest, and restricting to only those words that appear in at least 150 documents. Thus, the result of the co-occurrence of 26 words is organized around three clusters: the first related to “psychological” studies of interpersonal relationships between young students and adolescents (red), the second around clinical studies in workplaces between adults (green) and finally a third with the appearance of key words in a criminological perspective of sexual behavior, especially in the U.S. context (blue).

### 3.3. Authors and co-authorship analysis

The 3,384 documents were written by 6,064 different authors. Among all of them we can highlight nine for having written at least 10 of the documents contained in the Scopus database related to sexual harassment. The list consists of 7 women and 2 men, all of who are members of different North American universities. In addition, it is headed by Louise F. Fitzgerald of the University of Illinois with 26 records and 20 of them in the first stage (1978–2011), while Dorothy L. Espelage of the University of North Carolina is the most prolific in the last decade with 12 records (see Table 2). Precisely these two authors have participated in the two most cited articles: the first with 534 citations (Fitzgerald et al., 1988) and the second with 468 citations (Birkett et al., 2009).

**Table 2:** Leading authors by documents and periods.

Author	Sex, subject, university (country)	1978–2021	1978–2011	2012–2021
Fitzgerald, Louise F.	Woman, Psychology, University of Illinois (USA)	26	20	6
Stockdale, Margaret S.	Woman, Psychology, Indianapolis University (USA)	18	12	6
Wiener, Richard L.	Man, Psychology, University of Nebraska (USA)	17	10	7
Cortina, Lilia M.	Woman, Psychology, University of Michigan (USA)	16	9	7
Espelage, Dorothy I.	Woman, Education, Indianapolis of North Carolina (USA)	16	4	12
Dougherty, Debbie S.	Woman, Organizational Communication, University of Nebraska (USA)	13	8	5
Gutek, Barbara A.	Woman, Management and Organizations, University of Arizona (USA)	11	11	0
Magley, Vicki J.	Woman, Psychology, University of Connecticut (USA)	11	8	3
Pryor, John B.	Man, Psychology, University of Illinois (USA)	10	9	1

**Figure 3:**
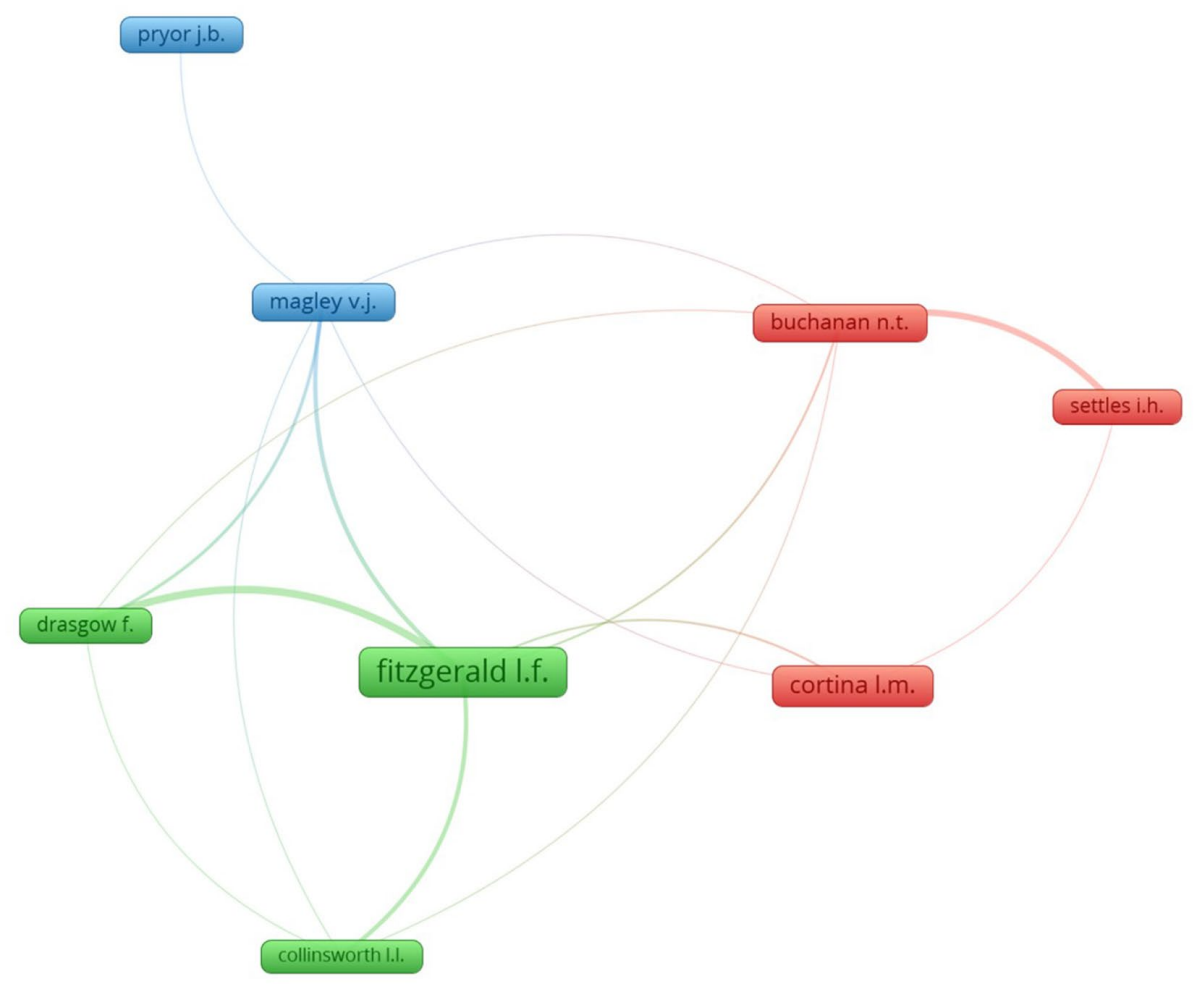
Analysis of co-authorship.

The analysis of co-authorship is presented in Figure 3, where we selected only those authors with at least five papers. This way, the sample was reduced to 61 authors, of whom only seven had co-authored a publication. The VOSviewer program obtained three clusters among the seven most prolific co-authors, the first and most relevant being that formed by Fitzgerald, Drasgow, and Collinsworth (green), the second by Buchanan, Settle, and Cortina (red) and the third by Magley and Pryor (blue). In any case, on one hand, the author Fitzgerald stands out for having a greater number of collaborations, especially with Drasgow and, on the other hand, Buchanan who has collaborated more times with Settles.

## 4. Conclusion

First of all, the trend of increasing interest in sexual harassment is evident in the analysis. This trend is especially prominent in the last 5 years. The explanation we propose focuses on the change in social awareness as a result of highly publicized cases (such as the Weinstein case), and the activity of social movements reporting this type of behaviour (#metoo and time’s up, among others).

As we mentioned in the theoretical part, interest in the subject has been diversifying, and the origin of the authors has broadened. We point out different reasons for that increase. We explain the increased attention in Sweden by relating it to a change in its policies, -for example, the change in Swedish regulations on sexual consent in 2018-. In the case of Spain, we consider that very mediatic cases, such as that of the “Manada” in 2016 in Pamplona (a group rape of a young woman perpetrated by five men), which have meant a rupture in the way of understanding sexual consent and the important feminist mobilizations on dates such as March 8 or November 25 have risen from this event. In both countries a similar situation can be seen in the way that, in Sweden, there is also a very mediatic case, -group rape of a minor in 2003- that gave rise to a controversial sentence and which years later lead to a legislative change on sexual consent. In Spain, a proposal of legislative change along the lines of the Swedish regulation has also been raised recently.

Secondly, the results also show a tendency of increased interest in sexual harassment concerning sexual violence, the workplace and gender studies. The analysis of the evolution of the keywords, therefore, suggests, that there is a clearer perception that sexual harassment is a type of violence, and that this violence is connected to gender inequalities, which implies an increase in research from this perspective. It would be necessary to make a more exhaustive review to know how many texts are situated in a feminist line of analysis, but the fact that the word gender appears so clearly already denotes a specific orientation in the studies.

As for the cluster analyses, the first of these, referring to keywords, suggests groupings between studies along various lines. One of them, marked in blue, would be the criminological perspective that focuses on analyzing the phenomenon in the U.S. context. This is a historical line if we take into account that the origin of the term first occurred in the USA and that this country is a pioneer when it comes to legislating on this type of violence. A cluster is also observed aimed at the analysis with a mainly psychological approach to the relationships that occur between students, young people and adolescents, -marked in red-. This cluster shows the importance of studying this phenomenon in the younger generations. In this sense, we are surprised that no words related to new technologies appear, especially considering that results on a European level show a high incidence of sexual harassment using the Internet in the younger groups of citizens (Cuenca-Piqueras et al., 2020). Indeed, it is more shocking if we take into account the recent studies reviewing the research about cyberviolence victimization in intimate or ex-intimate relationship contexts (Fernet et al., 2019).

Finally, in the cluster analysis on the authorship of the texts, the results suggest the different theoretical approaches presented at the beginning of the article. We part from the premise that the socio-cultural approach, which deals with gender-based power differences and is considered the most widely used or hegemonic, is shared by most authors and is, at least observed, in almost all research. In this sense, the cluster headed by Louise Fitzgerald, -who, according to O’Donohue et al. (1998) made one of the first and most widespread definitions of sexual harassment from a psychological approach-, together with Stockdale and Drasgow, -green cluster-, would work, as we mentioned, within the socio-cultural model and would also represent the studies on the organizational model, since they carry out pioneering analyses on harassment focused on labour sectors, working conditions, among others. On the other hand, the red cluster, composed of researchers such as Cortina and Buchanan, would also work on the sociocultural model but, on occasion, they carry out work focused on ethnic minorities, beginning to rely on the multidimensional model. Of course, this explanation has important limitations taking into account that most researchers vary, within the same field of study, from one type of theoretical approach to another depending on the group or social reality we analyze, and we also evolve in our analyses throughout our careers, but we do perceive in these clusters a general tendency towards the aforementioned approaches.

In short, it will be necessary to continue researching the scientific production on sexual harassment, on one hand, by dealing in greater depth with some areas of outstanding studies such as sexual harassment in the workplace or the association between harassment and violence, etc. and, on the other hand, by carrying out meta-analyses that provide us with an overview of the results of the studies over time.

## Author contributions

All authors listed have made a substantial, direct, and intellectual contribution to the work and approved it for publication.

## Conflict of interest

The authors declare that the research was conducted in the absence of any commercial or financial relationships that could be construed as a potential conflict of interest.

## Publisher’s note

All claims expressed in this article are solely those of the authors and do not necessarily represent those of their affiliated organizations, or those of the publisher, the editors and the reviewers. Any product that may be evaluated in this article, or claim that may be made by its manufacturer, is not guaranteed or endorsed by the publisher.
